# Primary care disaster management for extreme weather events, South Africa

**DOI:** 10.4102/phcfm.v14i1.3778

**Published:** 2022-12-19

**Authors:** Keshena Naidoo, Tawanda Manyangadze, Christian L. Lokotola

**Affiliations:** 1 Department of Family Medicine, College of Health Sciences, University of KwaZulu-Natal, Durban, South Africa; 2 Department of Geography, Faculty of Science, Bindura University of Science Education, Bindura, Zimbabwe; 3 Department of Family and Emergency Medicine, Stellenbosch University, Cape Town, South Africa

**Keywords:** climate change, flooding, primary care facility, disaster management, extreme weather

## Abstract

Primary health care facilities are at the forefront of helping communities affected by natural disasters. However, such facilities are also vulnerable to the effects of extreme weather events triggered by climate change. The April 2022 floods in the south-eastern region of South Africa claimed the lives of over 400 people, the loss of 16 000 houses and resulted in major damage to infrastructure. Most damage was localised in the eThekwini area in KwaZulu-Natal, which is the country’s third most populous city. This report describes the impact of the floods on primary health care facilities in eThekwini and their preparedness for extreme weather events.

**Contribution:**

Extreme weather events induced by climate change highlight the need for primary health care facilities to develop disaster management strategies that consider climate change.

## Background

On 11 April 2022, over 300 mm of rainfall occurred within a 24 h period in the south-eastern part of South Africa. Severe flooding and landslides caused by the heavy rainfall claimed the lives of over 400 people, destroyed 16 000 houses and resulted in disrupted water supplies and telecommunications.^1,2^ According to the South African Department of Health, there was an estimated R187 million worth of damage to health infrastructure, including 23 hospitals, 34 clinics, 3 community health centres and 5 office buildings.^3^ eThekwini, the country’s third most populous city, was the hardest hit.^5^ Water supply to major parts of the city was disrupted with repairs estimated to take months. Major roads were flooded and succumbed to sinkholes. Consequently, access to health facilities was affected.

There are global concerns about extreme weather events associated with climate change, and in particular, concerns about the readiness of health facilities to respond to such calamities.^3^ This report aims to describe the impact of the April 2022 floods on primary care facilities in the eThekwini district and their preparedness for extreme weather events. Data on the impact of the floods on health services were collected through document analysis and in-depth interviews with managers at two community health centres in eThekwini district that were affected by the flooding.

## Experience

Managers at health facilities in eThekwini district in the province of KwaZulu-Natal (KZN) were alerted to the extreme weather conditions through social media and news. Although government health facilities had some disaster preparedness plans to manage common disasters, extreme weather events were not specifically addressed. There was also no formal communication channel to facilitate sharing of information during the disaster. Hence, unforeseen challenges were experienced during and after the floods.

The heavy rainfall resulted in damage to roads, interrupted water supply and disrupted telecommunications. Staff and patients were unable to reach health facilities because of flooding of main roads as well as mudslides. However, emergency services continued to operate by extending the working hours of the skeleton staff on site. At some facilities all health services had to be deferred until conditions improved. One community health centre had to be closed after several departments were flooded. Loose sand from an on-site construction area washed through several departments in the facility, resulting in damage to equipment and the building. This facility has remained closed for more than 4 months since the floods while refurbishment continues. The staff were subsequently redeployed to other facilities.

Another challenge was the disruption of the water supply, in some areas for up to 8 weeks. Although water tankers were dispatched from the municipality to health facilities, there was an initial delay. Fortunately, donations of drinking water by non-governmental organisations and private individuals provided some relief to the clinics. Health providers were advised to be vigilant for water-borne diseases, but fortunately there was no outbreak of any such illnesses.

Displaced communities lost not only their homes, but their medication and medical devices as well. Mobile health teams were tasked with visiting centres accommodating displaced people, and providing health screening and dispensing medication. Additional medication had not been budgeted for, resulting in treatment disruption for chronic patients.

The floods highlighted weaknesses in the municipality’s infrastructure such as stormwater drainage and roads. Furthermore, the effects of heavy rainfall were exacerbated by the expansion of human settlements and the blockage of stormwater drains by plastic pollution.^4^

Some of the disaster management strategies at healthcare facilities included on-site generators in the event of power outages. Ironically, these generators consume diesel – a fossil fuel contributing to climate change.

## Reflection

There is an evident need for early warning systems and for improved communication channels to ensure that staff at health facilities can anticipate and prepare for extreme weather events such as the recent flooding in KZN. Although both the South African Weather Service and eThekwini municipality issued early warnings for severe weather events, the information was not cascaded down to health facility managers.^5^ eThekwini was one of the first cities in South Africa to develop an early warning system for flooding with real-time communication with communities vulnerable to flooding such as informal settlements in low-lying areas. During the April 2022 floods communities in five high-risk areas were advised to evacuate, thereby avoiding loss of life. Wider access to such early warning systems could assist health facilities to anticipate and prepare for extreme weather conditions.

Even when facilities are alerted to imminent extreme weather, there are no clear directives on what actions to take. Current disaster plans in the Department of Health do not specifically address extreme weather conditions. Climate change is an area that has been ignored by health systems, in both mitigating the effect of health systems on global warming and preparing for the impact of climate change on population health. In the light of the recent flooding, greater attention should be focussed on how primary health care can tackle the issue of climate change. Furthermore, funding should be available to mobilise resources when required such as purchasing water and additional medication during disasters. Existing disaster plans have underestimated the resources required to deliver essential health services during extreme weather conditions. Facilities could mitigate the effect of water and electricity interruptions by harvesting and storing rainwater, and utilising generators with hybrid power supplies.

Primary health facilities are dependent on functional communication, transportation and access to potable water in order to deliver health services to the communities they serve. Therefore, it is essential for the health sector to engage with other sectors such as water and sanitation, transportation services and social services. Furthermore, the development of any meaningful disaster plan should involve planning and coordination with the relevant stakeholders.

## Conclusion

Primary health care facilities are as vulnerable to the impact of climate change as the communities they serve. The healthcare sector must consider extreme weather events and develop communication channels around early warning systems. Furthermore, the development of disaster management plans must include intersectoral collaboration and emergency funding.
